# Artemisia pollen-induced allergic rhinitis in mice: multi-omics dissection of local and systemic molecular alterations

**DOI:** 10.3389/falgy.2026.1835987

**Published:** 2026-07-08

**Authors:** Jiaoni Chi, Lin Chen, Yuxuan Jin, Jin-Lyu Sun, Yajie Xu

**Affiliations:** 1 Department of Allergy, State Key Laboratory of Complex Severe and Rare Diseases, Peking Union Medical College Hospital, Chinese Academy of Medical Sciences & Peking Union Medical College, Beijing, China; 2 Research Unit of New Techniques for Live-cell Metabolic Imaging, Chinese Academy of Medical Sciences, Beijing, China; 3 Department of Trauma-Emergency & Critical Care Medicine, Shanghai Fifth People’s Hospital, Fudan University, Shanghai, China

**Keywords:** allergic rhinitis, metabolomics, mugwort, multi-omics, transcriptomics

## Abstract

**Background:**

Mugwort (*Artemisia vulgaris*) is a predominant aeroallergen for allergic rhinitis (AR) in northern China. However, the molecular changes linking local nasal mucosal inflammation with systemic alterations remain incompletely understood. This study aimed to explore the cross-level regulatory network in a mouse model of mugwort-induced AR by combining nasal mucosal transcriptomics and serum metabolomics.

**Methods:**

BALB/c mice were sensitized with mugwort extract and then challenged intranasally to establish an AR model. Nasal mucosal tissues were collected for RNA sequencing, and serum samples from the same cohort were subjected to non-targeted metabolomic analysis. Transcription factor (TF)-associated analysis, pathway enrichment analysis, and integrative multi-omics analysis were used to identify candidate regulatory factors and pathways involved in mugwort-induced allergic inflammation. Targeted validation of arginine-related changes was performed by measuring serum Arg1 and L-arginine levels, followed by *ARG2* knockdown analysis in BEAS-2B epithelial cells after mugwort extract stimulation.

**Results:**

Transcriptomic analysis revealed a clear Th2-type immune response in the model group, with significant upregulation of *Il13*, *Arg1*, *Ccl24* and *Il6*. In addition, many downregulated genes were enriched in pathways related to ciliary function and epithelial differentiation, accompanied by suppression of structural genes such as *Krt25* and *Krt71*. Gene set enrichment and TF-associated analyses further highlighted cytokine-mediated signaling and potential upstream regulators, including *Fos*, *Batf*, and *Mafb*. Serum metabolomics showed increased 1-methylhistamine and enrichment of arachidonic acid metabolism in mugwort-treated mice. Integrated analysis of the two omics datasets further pointed to arginine-related metabolism as a shared altered pathway. Consistently, targeted assays showed increased serum Arg1 levels and decreased serum L-arginine concentrations in the mugwort group. In BEAS-2B cells, *ARG2* knockdown attenuated the induction of *IL6* and *CCL26* after mugwort extract stimulation.

**Conclusion:**

Together, our findings indicate that mugwort-induced AR is accompanied by coordinated local and systemic changes, including immune activation, epithelial/ciliary dysfunction, and serum metabolic remodeling. The arginine-related alterations supported by both omics analysis and targeted validation provide a potential link between nasal mucosal inflammation and systemic metabolic changes in mugwort allergy.

## 1 Introduction

Allergic rhinitis (AR) is a chronic inflammatory disease triggered by inhaled allergens and characterized by a Th2-dominant immune response, primarily involving the nasal mucosa (1–3). Typical symptoms include nasal congestion, rhinorrhea, nasal itching, and paroxysmal sneezing. AR affects patients’ sleep quality, daytime functioning, and work productivity, and is also closely associated with other atopic disorders, including asthma, atopic dermatitis, and sinusitis (4). The global prevalence of AR is estimated to range from 10% to 40%, with a continuous upward trend in recent years (2).

Sensitization patterns may differ due to geographical location and environmental exposure (5, 6). In northern China, mugwort pollen represents the most predominant aeroallergen for seasonal AR (7). Epidemiological surveys have shown that among patients seeking care for respiratory allergies during the late summer and autumn, the sensitization rate to mugwort exceeds 50%, considerably higher than that for other weed pollens such as Humulus and Ambrosia. Beyond nasal symptoms, mugwort allergy is closely associated with the development and exacerbation of asthma, emphasizing its clinical significance (8).

High-throughput omics technologies have been widely used for systematically deciphering the pathogenesis of AR (9, 10). Existing studies have employed multi-omics approaches in mouse models of asthma induced by house dust mite extract, revealing the reprogramming features of lipid metabolism in lung tissue (11). In contrast, investigations on the multi-omics level of the AR model induced by mugwort pollen remain relatively scarce. Nasal mucosa transcriptomics analysis enables the capture of local immune activation, epithelial remodeling, and changes in cytokine-chemokine networks. Serum metabolomics can comprehensively reflect the functional state of systemic metabolic remodeling, encompassing immune activation, oxidative stress, and host-microbiota interactions (12, 13). However, the perspectives provided by a single omics data are ultimately limited. Through multi-omics integration analysis, coordinated gene-metabolite modules can be uncovered, and potential mechanism axes connecting local mucosal inflammation with systemic biochemical alterations can be identified.

In this study, we established an AR model with mugwort crude extract in BALb/c mice and performed transcriptomic analysis of the nasal mucosa along with untargeted metabolomic profiling of serum. We further characterized the coupled molecular network related to AR by multi-omics integration analysis. Our works aimed to identify the transcriptional landscape of the nasal mucosa and systemic metabolic alterations, and to elicit gene–metabolite interactions that may contribute to AR pathogenesis, providing potential biomarkers or therapeutic targets.

## 2 Materials and methods

### 2.1 Animals

Specific pathogen-free female BALB/c mice (8 weeks old, approximately 22 g) were purchased from Shanghai Shengchang Bio-tech Co., Ltd and acclimated under a 12/12 h light/dark cycle at 25 ℃ for one week prior to sensitization. Studies were approved by the Institutional Animal Ethics Committee (Approval No. SR20250701).

### 2.2 Mouse sensitization

Mice were randomly assigned to a control group (*n* = 6) and a mugwort-induced AR group (*n* = 6). A sensitization-challenge protocol was used to establish the AR model (14, 15). The AR group received an intraperitoneal injection of 200 μL solution containing 100 μg mugwort crude extract (Stallergenes Greer) and 1 mg aluminum hydroxide adjuvant on days 0 and 7. Control group received an equal volume of PBS. From days 14 to 17, AR mice were challenged intranasally once daily with 1 mg mugwort extract in 20 μL PBS for four consecutive days. The experiment was terminated 24 h after the final challenge. Mice were then euthanized for sample collection. For each mouse, both nasal mucosal tissue and serum were collected at the same endpoint. The same cohort of mice was used for nasal mucosal RNA-seq and serum metabolomic profiling.

### 2.3 Histological analysis and inflammation scoring

Nasal mucosa tissue was fixed, paraffin-embedded, sectioned, and stained with hematoxylin and eosin (H&E) following standard protocols. Histological inflammation was semi-quantitatively scored as previously described with minor modifications (16). The score was defined as follows: 0, no obvious inflammatory cell infiltration or edema; 1, mild focal inflammatory cell infiltration; 2, moderate inflammatory cell infiltration with mucosal/submucosal edema; 3, severe or diffuse inflammatory cell infiltration with obvious mucosal structural changes. The fields of view used for inflammation scoring were randomly selected for each mouse.

### 2.4 Serum preparation and ELISA measurement

Blood samples were collected under anesthesia 24 h after final challenge and clotted at room temperature for 2 h. Serum was then separated by centrifugation at 3,000 rpm for 15 min and stored at −80℃ until analysis. Commercial ELISA kits were used to determine serum concentrations of total IgE (Shanghai Jonlnbio) and Arg1 (Shanghai Jonlnbio). All procedures were carried out according to the manufacturer's instructions. Absorbance was measured at 450 nm using a microplate reader (BioTek Instruments, Inc.).

### 2.5 Measurement of serum L-arginine levels

Serum L-arginine levels in PBS and mugwort groups were measured using a commercial arginine assay kit (Beijing Solarbio) according to the manufacturer's instructions. Briefly, serum samples were pretreated with the extraction reagents provided in the kit and centrifuged to obtain the supernatant for analysis. L-arginine was detected using a visible spectrophotometric colorimetric assay based on the reaction of arginine with *α*-naphthol and sodium hypochlorite under alkaline conditions, which generates a colored complex. The absorbance of the reaction product was measured at 525 nm using a microplate reader (BioTek Instruments, Inc.).

### 2.6 Collection and preservation of nasal mucosa tissue

Mice nasal mucosa tissues were collected as previously described with minor modifications (17). Briefly, the skin over the heads of the mice was bluntly dissected to expose the nasal structures. The nasal bone was then gently lifted upward along the nostrils using fine pointed forceps to open the nasal cavity. Soft tissues overlying the palate were carefully separated to expose the floor of the nasal cavity. To fully expose the posterior nasal cavity, the maxillary teeth together with the alveolar bone on both sides were cut and everted laterally. The palatal bone plate located at the base of the posterior airway was then pried open to expose the entire posterior choana. Subsequently, the nasal mucosa and associated cartilage were carefully dissected from both the superior and inferior aspects of the nasal cavity. Mucosal tissues attached to the cartilage were further cleaned to ensure complete removal. The collected nasal mucosa tissues were immediately placed in RNAlater solution (Thermo Fisher Scientific, Waltham, MA, USA) for preservation and subsequent transcriptomic analyses.

### 2.7 Nasal mucosa transcriptomic analysis

Nasal mucosal RNA-seq was performed using tissues from mice treated with PBS (*n* = 6) and mice exposed to mugwort (*n* = 6). Total RNA of mice nasal mucosa was extracted using the TRIzol reagent (Invitrogen, CA, USA) according to the manufacturer's protocol. RNA purity and quantification were measured using the NanoDrop 2000 spectrophotometer (Thermo Scientific, USA). RNA integrity was assessed using the Agilent 2100 Bioanalyzer (Agilent Technologies, Santa Clara, CA, USA). Qualified RNA samples were used for transcriptome library construction and sequenced on an Illumina Novaseq X Plus platform. Raw FASTQ reads underwent quality control to remove low-quality reads, yielding clean reads for subsequent analysis. Clean reads were aligned to the mouse reference genome, and gene expression was quantified to generate an expression matrix for each sample. This matrix was then used for downstream differential expression and functional enrichment analyses.

### 2.8 Untargeted metabolomic profiling of serum

Serum metabolomic profiling was performed using serum samples from the same PBS-treated (*n* = 6) and mugwort-treated (*n* = 6) mice that were subjected to nasal mucosal RNA-seq. Frozen serum samples were thawed on ice before being tested. Each sample was mixed with internal standard solution and extracted with pre-cooled methanol and acetonitrile (2:1, vol/vol), followed by vortex mixing and overnight incubation at −40 ℃. The extracts were centrifuged at 4 ℃ (12,000 rpm) for 20 min, and the supernatants were collected for LC–MS/MS analysis. After a second centrifugation step, the final supernatants were filtered through 0.22 μm microfilters and transferred to LC vials. Quality control (QC) samples were prepared by mixing aliquots of all samples to generate a pooled sample.

Metabolic profiling was performed on an ACQUITY UPLC I-Class plus system (Waters Corporation, Milford, USA) fitted with Q-Exactive mass spectrometer equipped with a heated electrospray ionization source (Thermo Fisher Scientific, Waltham, MA, USA) in both positive and negative ion modes. An ACQUITY UPLC HSS T3 column (1.8 μm, 100 × 2.1 mm) was employed in both modes. The mobile phases consisted of water containing 0.1% formic acid and acetonitrile under a gradient elution program. Mass spectrometry data were acquired in Full MS/dd-MS2 mode over a scan range of m/z 70–1050.

Raw data were processed using XCMS V4.5.4 for baseline filtering, peak identification, integration, retention time (RT) correction, peak alignment, and normalization. Compound identification was performed based on RT, accurate mass, MS/MS fragments, and isotopic distribution by matching against The Human Metabolome Database (HMDB), Lipidmaps (v2.3), METLIN, and self-built databases for analysis. Annotation confidence levels were assigned according to the platform criteria, with Level 1 defined as RT deviation within ±0.3 min and fragmentation score ≥45. Level 2 defined as RT deviation within ±0.3 min but lower fragmentation scores. Detailed annotation information for key metabolites is provided in Supplementary Table S1.

Quality control (QC) samples were prepared by pooling equal aliquots of all serum extracts and were used to monitor analytical stability. To ensure data quality, features with a relative standard deviation (RSD) greater than 30% in QC samples were excluded. In addition, features with more than 50% missing values in any group were removed. The remaining missing values were imputed using half of the minimum ion intensity, followed by normalization and log2 transformation for downstream differential metabolite screening and pathway analysis.

### 2.9 Integrated transcriptomic-metabolomic analysis

For the integrated transcriptomic–metabolomic analysis, differentially expressed genes (DEGs) were defined using thresholds of *q* value <0.05 and fold change >2.0 or <0.5. Differential metabolites were selected according to the metabolomic filtering criteria described above. Associations between DEGs and differential metabolites from the same experimental cohort were evaluated using Spearman correlation analysis. Correlations with *p* value <0.05 were considered significant.

### 2.10 Statistical and bioinformatic analysis

Statistical analysis was performed using GraphPad Prism software and R software. Comparisons between two groups were conducted using an unpaired Student's *t*-test. For comparisons among multiple groups, one-way ANOVA followed by multiple-comparison tests was performed. A two-sided *p* value <0.05 was considered statistically significant.

For transcriptomic data, principal component analysis (PCA) was performed to assess clustering and biological duplication among samples. Differential expression analysis was conducted using the DESeq2. Genes with *q* < 0.05 and log2(FoldChange) >1 were defined as DEGs. Based on the hypergeometric distribution, Gene ontology (GO) and Kyoto Encyclopedia of Genes and Genomes (KEGG) enrichment analysis of DEGs were performed using R (v 3.2.0). For GO and KEGG enrichment analyses, the background gene set consisted of all genes detected and quantified in the RNA-seq datasets with available functional annotations. Gene set enrichment analysis (GSEA) was conducted to identify significantly enriched functional categories and pathways.

For metabolomic data, the matrix was imported in R to carry out PCA to observe the overall distribution among the samples and the stability of the analytical process. Orthogonal Partial Least-Squares-Discriminant Analysis (OPLS-DA) and Partial Least-Squares-Discriminant Analysis (PLS-DA) were utilized to distinguish the metabolites that differed between AR group and control group. Variable Importance in Projection (VIP) values obtained from the OPLS-DA model were used to rank the overall contribution of each metabolite to group discrimination. Differential metabolites were selected using the criteria described in the metabolomic analysis, including *p* value <0.05 and fold change ≥1.2 or ≤1/1.2. For metabolite pathway enrichment analysis, the background metabolite set consisted of all annotated metabolites retained after QC filtering and mapped to pathway databases.

## 3 Results

### 3.1 Establishment and validation of the mugwort-induced AR model

**Figure 1:**
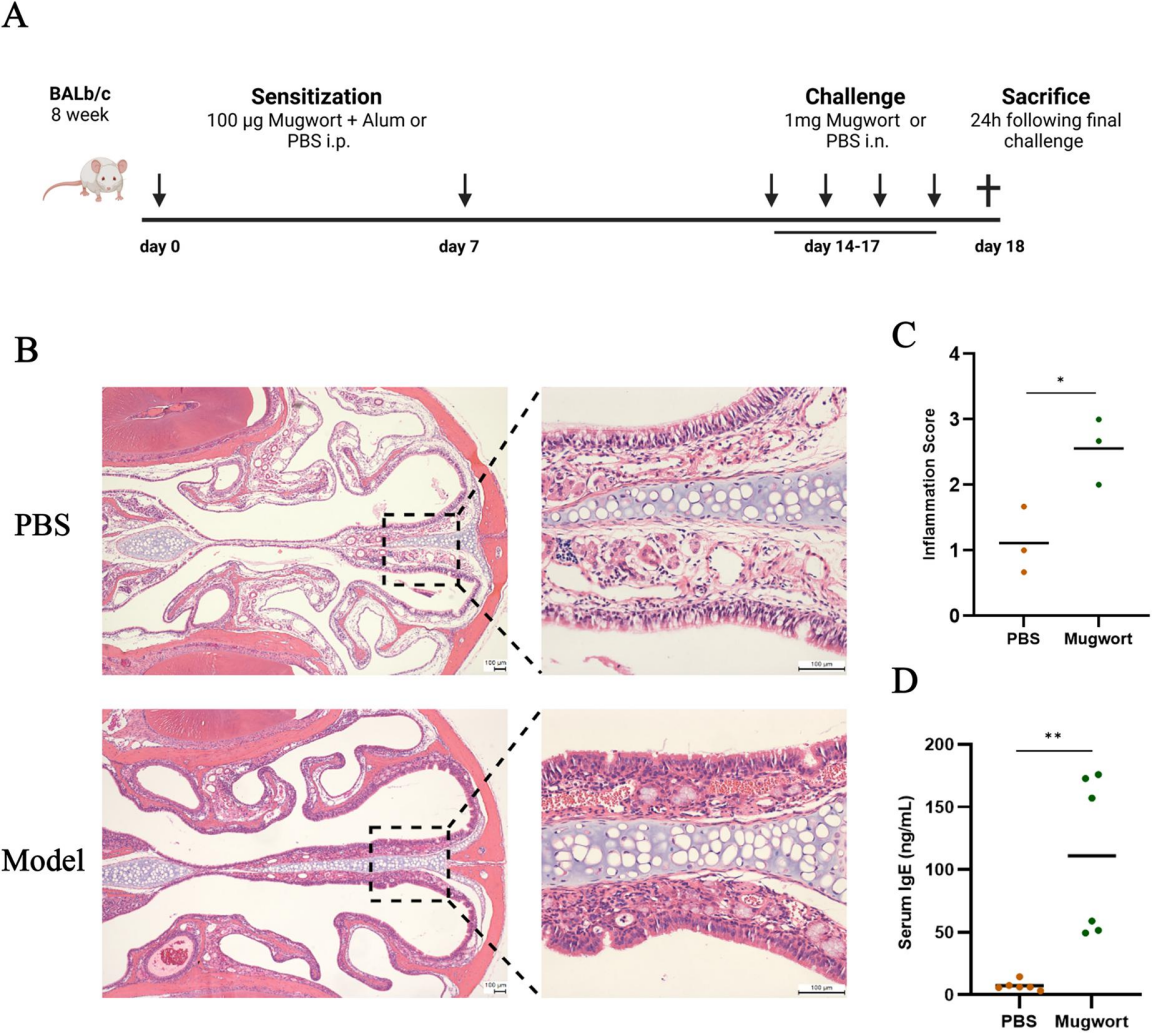
Establishment and validation in a mouse model of mugwort-induced AR. **(A)** Schematic diagram of the sensitization and challenge protocol. Created in BioRender. Jiao, J. (2026) https://BioRender.com/z4xptk0. **(B)** Representative H&E staining of nasal mucosa tissues from PBS and mugwort groups, *n* = 3/group for histological analysis. For each mouse, three randomly selected non-overlapping fields were evaluated (50×, scale bar: 100 μm; 200×, scale bar: 100 μm). **(C)** Semi-quantitative histological inflammation score based on inflammatory cell infiltration, mucosal/submucosal edema, and overall mucosal structural changes using a 0–3 scale: 0, none; 1, mild; 2, moderate; 3, severe. For each mouse, three randomly selected non-overlapping fields were scored, and the mean value was used as the inflammation score for that mouse; *n* = 3/group. **(D)** Serum total IgE levels in the PBS and mugwort groups measured by ELISA, *n* = 6/group. Data are presented as the mean. Statistical significance was determined by unpaired Student's *t*-test. *p < 0.05, **p < 0.01, ***p < 0.001, ****p < 0.0001.

To investigate the biological mechanisms of AR induced by mugwort, we established a murine model by systemic sensitization followed by nasal challenge (Figure 1A). Histopathological examination by H&E staining revealed obvious lamina propria edema and massive inflammatory cell infiltration in the nasal mucosa of the mugwort group (Figure 1B). Semi-quantitative histological assessment showed that the mugwort group had markedly higher inflammation scores than the PBS control group (Figure 1C). Additionally, serum total IgE levels in the mugwort group were significantly increased compared to the control group (Figure 1D). These findings support the successful establishment of mugwort-induced allergic inflammation in mice.

### 3.2 Transcriptomic remodeling of the nasal mucosa

**Figure 2:**
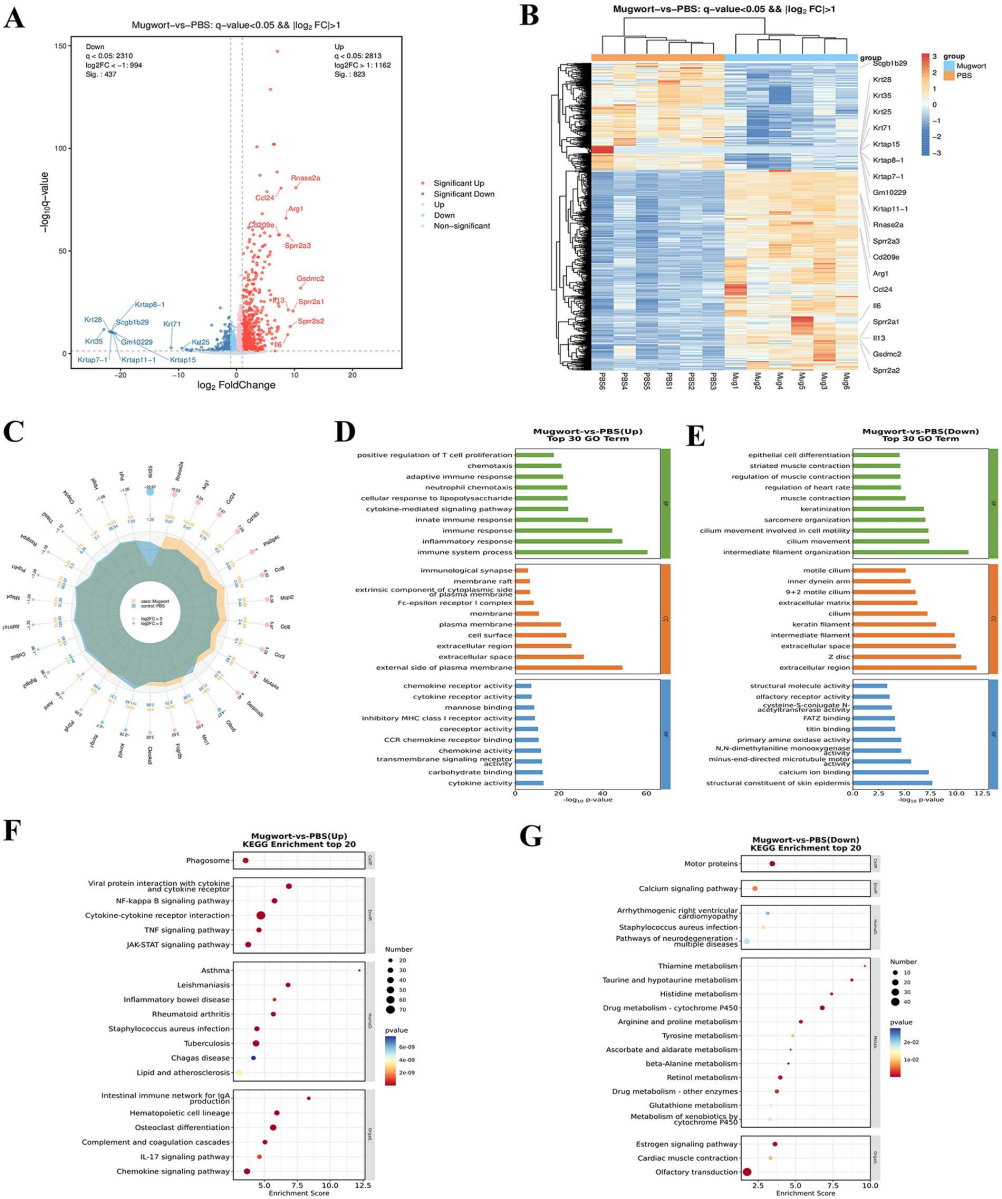
**Transcriptomic landscape of the nasal mucosa in mugwort-induced AR. (A)** Volcano plot showing DEGs between the mugwort and PBS groups. Red and blue dots indicate significantly upregulated and downregulated genes, respectively. **(B)** Clustering heatmap of representative DEGs in nasal mucosal tissues. **(C)** Radar plot showing the expression changes of representative DEGs in nasal mucosal tissues. **(D,E)** GO enrichment analysis performed separately for the upregulated and downregulated genes. **(F,G)** KEGG enrichment analysis for the top 20 upregulated and downregulated genes. DEGs were defined as genes with *q* < 0.05 and log_2_(FoldChange) >1. GO and KEGG enrichment results are revealed for significantly enriched terms/pathways.

To clarify local molecular alterations of AR, we conducted RNA-seq analysis on nasal mucosa tissues. PCA of the RNA-seq data showed separation between PBS and mugwort-treated nasal mucosal samples, indicating distinct transcriptional profiles between the two groups (Supplementary Figure S1A). Differential expression analysis revealed distinct transcriptional remodeling in mugwort group, with numerous significantly upregulated and downregulated genes (Figures 2A,B). Among the upregulated genes, representative Th2 and inflammation-related factors, such as *Il13*, *Ccl24*, and *Arg1*, were prominently increased (Figures 2A–C).

GO enrichment analysis further indicated that the downregulated genes were mainly enriched in pathways related to motile cilia and epithelial cell differentiation, while the upregulated genes were enriched in immune and cytokine-related processes (Figures 2D,E). Consistently, the heatmap results showed reduced expression of multiple structural or epithelial-related genes, including members of the *Krt* family such as *Krt2*5 and *Krt71* (Figure 2B). KEGG analysis further supported the enrichment of inflammation-related pathways, including asthma, JAK-STAT signaling, and IL-17 signaling pathway (Figures 2F,G). Taken together, these results indicate that mugwort stimulation induces a strong inflammatory transcriptional response in nasal mucosa, accompanied by the inhibition of transcriptional programs related to cilia and epithelial homeostasis.

### 3.3 Transcription factor (TF)-associated regulatory networks in AR progression

**Figure 3:**
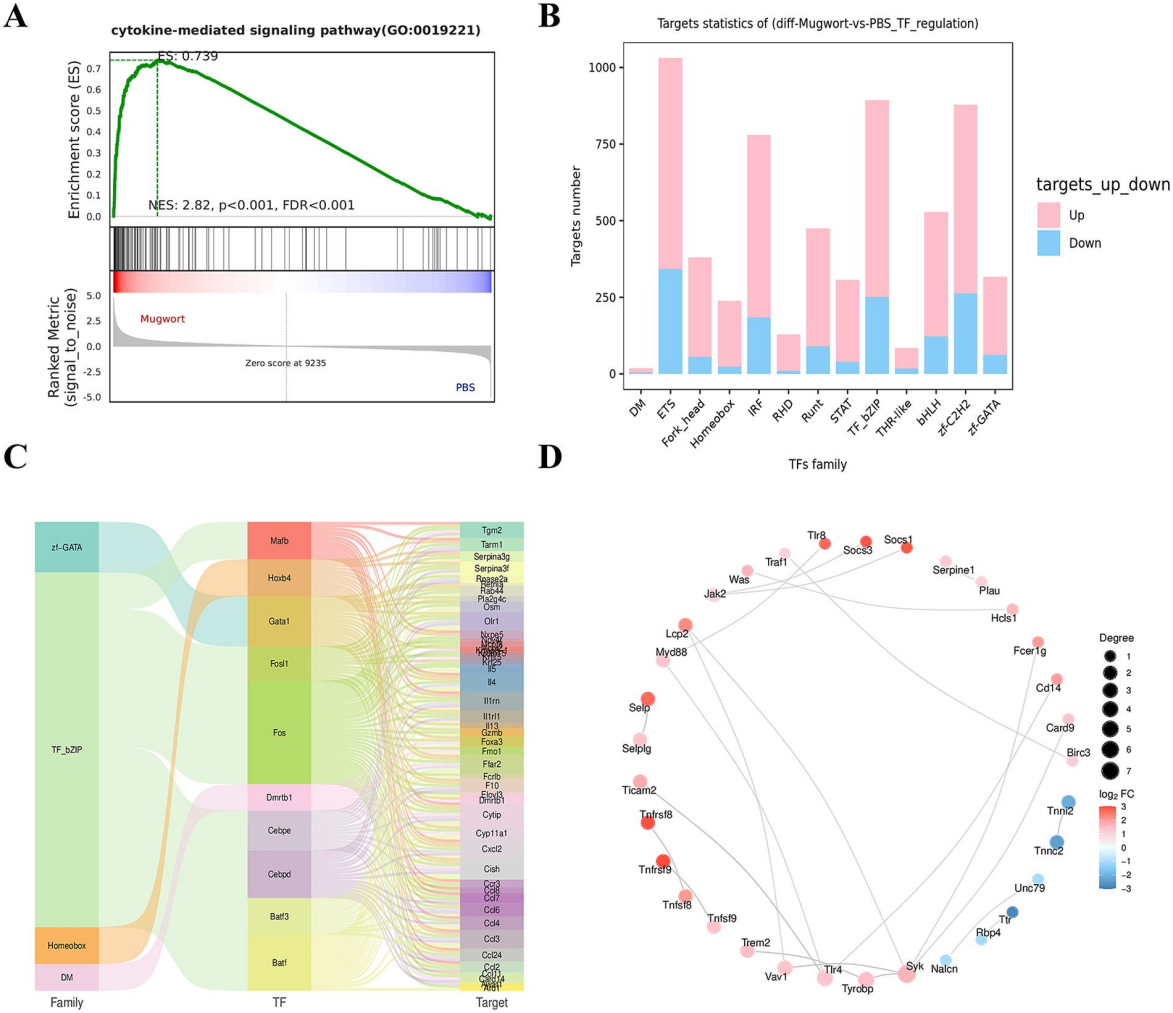
Cytokine-associated transcriptional programs and regulatory networks in mugwort-induced AR. **(A)** Gene set enrichment analysis (GSEA) showing enrichment of the cytokine-mediated signaling pathway in the Mugwort-vs-PBS comparison. The green curve represents the running enrichment score (ES) across the ranked gene list. A positive normalized enrichment score (NES) indicates that this gene set was enriched toward genes upregulated in the mugwort group. **(B)** Distribution of transcription factor (TF) target enrichment among differentially expressed genes (DEGs), showing the numbers of upregulated and downregulated target genes across representative TF families. **(C)** Sankey diagram of predicted TF regulatory relationships in the Mugwort-vs-PBS comparison. The three columns from left to right indicate TF families, differentially expressed TFs, and predicted differentially expressed target genes. The connecting bands represent family-TF-target relationships. For visualization, when the total number of differential TFs or target genes exceeded 100, the top 10 TFs and target genes ranked by absolute log_2_(FoldChange) were displayed. **(D)** Circular protein–protein interaction (PPI) network of the top 30 DEGs in the Mugwort-vs-PBS comparison. According to log_2_(FoldChange), red nodes represent upregulated genes, and blue nodes represent downregulated genes. Node size indicates the number of connected genes, and edges represent protein-protein associations.

To better understand the regulatory mechanisms underlying these transcriptional changes, we turned to TF-associated network analysis. GSEA showed enrichment of the cytokine-mediated signaling pathway in the mugwort group (NES = 2.82, *p* ＜ 0.001, FDR ＜ 0.001) (Figure 3A). The TF target statistics and Sankey diagram suggested that several TF families, as well as representative transcription factors including *Fos*, *Batf*, and *Mafb*, might be involved in the regulation of downstream inflammatory genes (Figures 3B,C). Moreover, protein–protein interaction network analysis further pointed to hub-like interactions among inflammation-related genes (Figure 3D). Together, these findings pointed to a coordinated transcriptional response in mugwort-induced AR, marked by the involvement of candidate upstream TFs and co-expressed modules tied to the allergic phenotype.

### 3.4 Serum metabolic profiling reveals pro-inflammatory metabolite alterations

**Figure 4:**
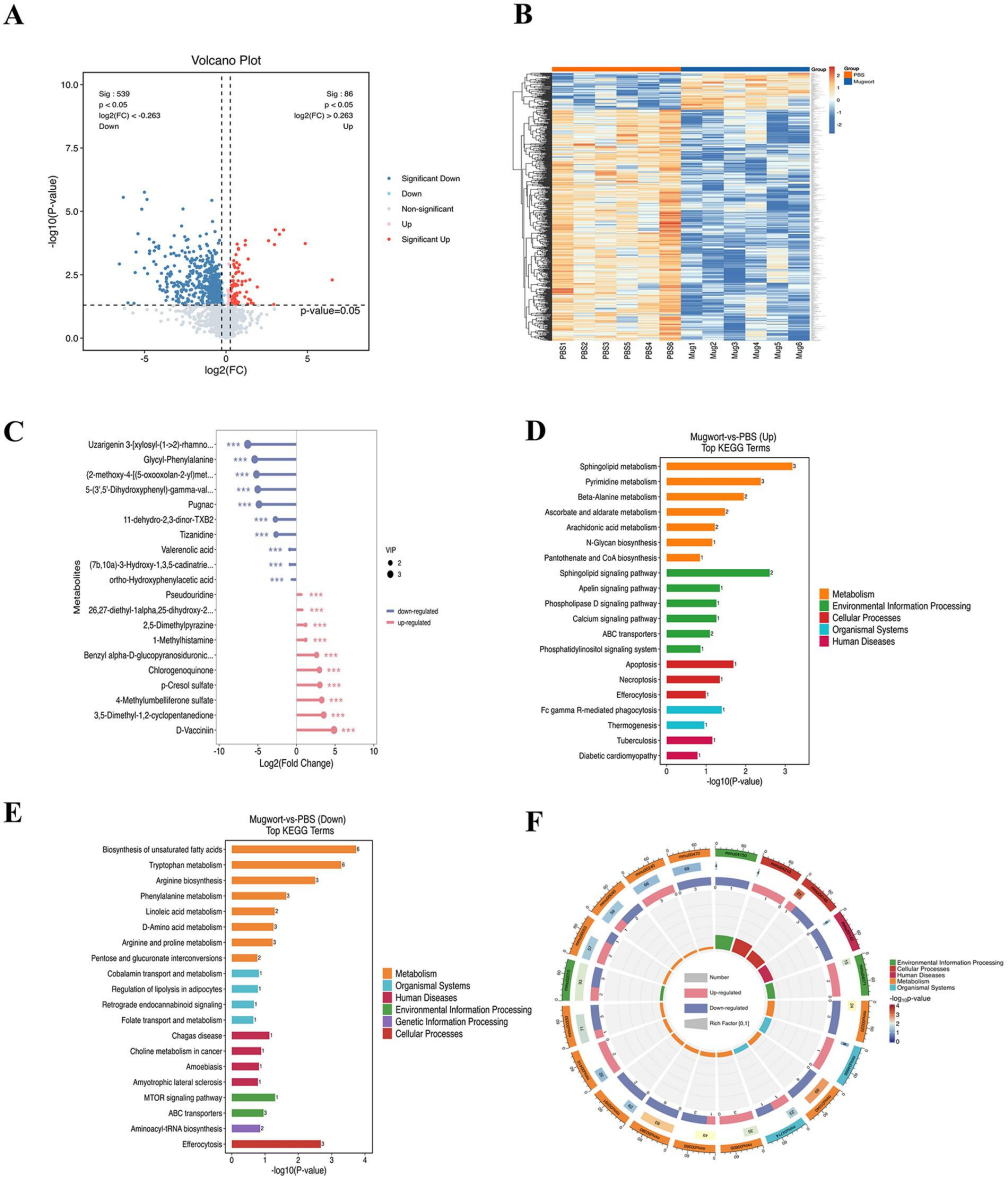
Serum metabolomic profiling reveals differential metabolites and enriched metabolic pathways in mugwort-induced AR mice. **(A)** Volcano plot demonstrating the differential metabolites between mugwort and control groups. Red dots indicate upregulated metabolites, and blue dots indicate downregulated metabolites. **(B)** Hierarchical clustering heatmap displaying serum metabolic shifts between the PBS and mugwort groups. Color from blue to red represents low to high metabolite abundance, with red denoting higher abundance of differential metabolites. **(C)** Lollipop plot depicting the relative log_2_(FoldChange) of the top 10 upregulated and downregulated differential metabolites (ranked by *p*-value) in the Mugwort-vs-PBS comparison. **(D,E)** KEGG enrichment analysis performed separately for upregulated and downregulated differential metabolites. **(F)** Circular visualization of KEGG pathway classification and enrichment of differential metabolites in the Mugwort-vs-PBS comparison. Differential metabolites were identified using the criteria VIP >1 and *p* < 0.05. *p < 0.05, **p < 0.01, ***p < 0.001, ****p < 0.0001.

Non-targeted metabolomics analysis on serum samples was conducted for the purpose of investigate the systemic metabolic changes associated with local nasal inflammation. PCA of the serum metabolomic data showed group-level separation between PBS and mugwort-treated mice (Supplementary Figure S1B). As shown in the volcano plot and hierarchical clustering heatmap, the metabolic profiles of the mugwort group showed clear shifts compared with controls. (Figures 4A,B). Among the annotated metabolites, 1-methylhistamine was elevated in the mugwort group (Figure 4C). The annotation confidence and supporting spectral information for this metabolite are provided in Supplementary Table S1. KEGG enrichment analysis revealed that the differential metabolites were enriched mainly in the arachidonic acid metabolism and tryptophan metabolism (Figures 4D,E). Further pathway-level visualization indicated that extensive metabolic disturbances across multiple biological systems (Figure 4F). These findings suggest that the AR induced by mugwort is accompanied by systemic metabolic remodeling in the serum, particularly involving histamine-related metabolites and inflammatory lipid metabolism.

### 3.5 Integrated transcriptomic-metabolomic analysis highlights arginine-related pathway alterations

**Figure 5:**
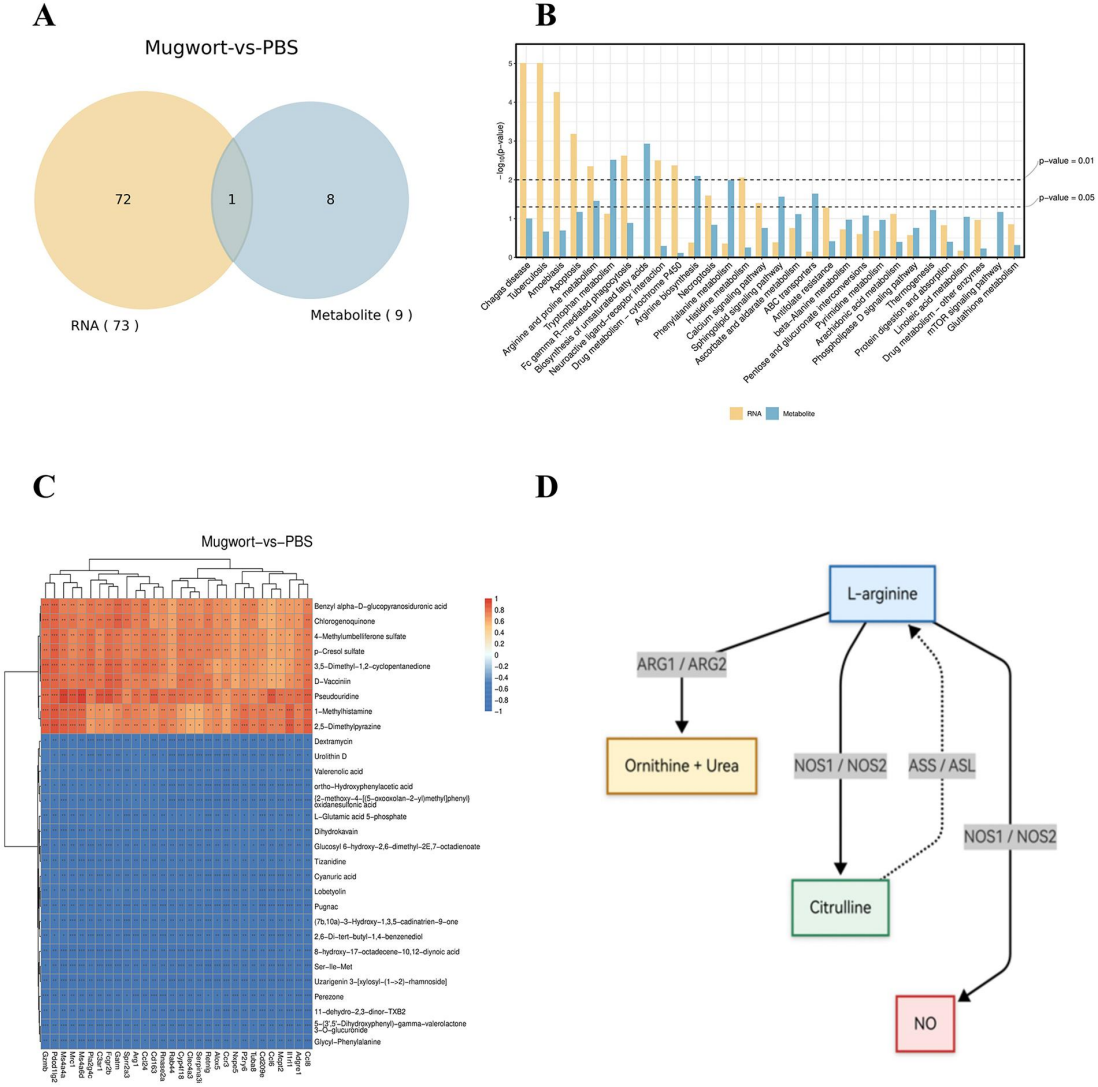
Integrated transcriptomic–metabolomic analysis highlights arginine-related pathway alterations in mugwort-induced allergic inflammation. **(A)** Venn diagram indicating overlap between RNA-derived and metabolite-derived KEGG pathway sets. **(B)** Joint enrichment analysis summarizing a shared set of arginine metabolism between the two omics layers. **(C)** Spearman correlation heatmap of the top DEGs and differential metabolites in the Mugwort-vs-PBS comparison. The top 30 features from each omics dataset were selected based on *p* value ranking. Red and blue indicate positive and negative Spearman correlations, respectively. *p < 0.05, **p < 0.01, ***p < 0.001. **(D)** Simplified schematic of intracellular arginine metabolism. ARG, arginase; ASS, argininosuccinate synthetase; ASL, argininosuccinate lyase; NOS, nitric oxide synthase.

To explore potential cross-omics associations, we performed an integrated analysis of the nasal mucosal transcriptomic data and serum metabolomic profiles. Overlap analysis of KEGG pathways indicated that arginine-related metabolism was shared between the transcriptomic and metabolomic datasets (Figure 5A). Joint enrichment analysis supported the involvement of this pathway across the two omics datasets (Figure 5B). Spearman correlation analysis showed associations between representative DEGs and differential metabolites, including inflammation-related genes and histamine-related metabolites such as 1-methylhistamine (Figure 5C). This pathway mapped 10 gene hits (*Aldh1b1*, *Aoc1*, *Aoc1l2*, *Arg1*, *Arg2*, *Ckm*, *Ckmt2*, *Cndp1*, *Gatm*, *Maob*) and 4 metabolite hits (L-arginine, spermidine, homocarnosine, and L-glutamic acid 5-phosphate). Although the enriched KEGG term was annotated as arginine and proline metabolism, the mapped molecules in our datasets were mainly related to the arginine branch. A simplified schematic of intracellular arginine metabolism is shown in Figure 5D. Together, these results suggest pathway-level convergence of transcriptomic and metabolomic alterations related to arginine metabolism in mugwort-induced allergic inflammation.

### 3.6 Targeted validation of arginine-related metabolic alterations

**Figure 6:**
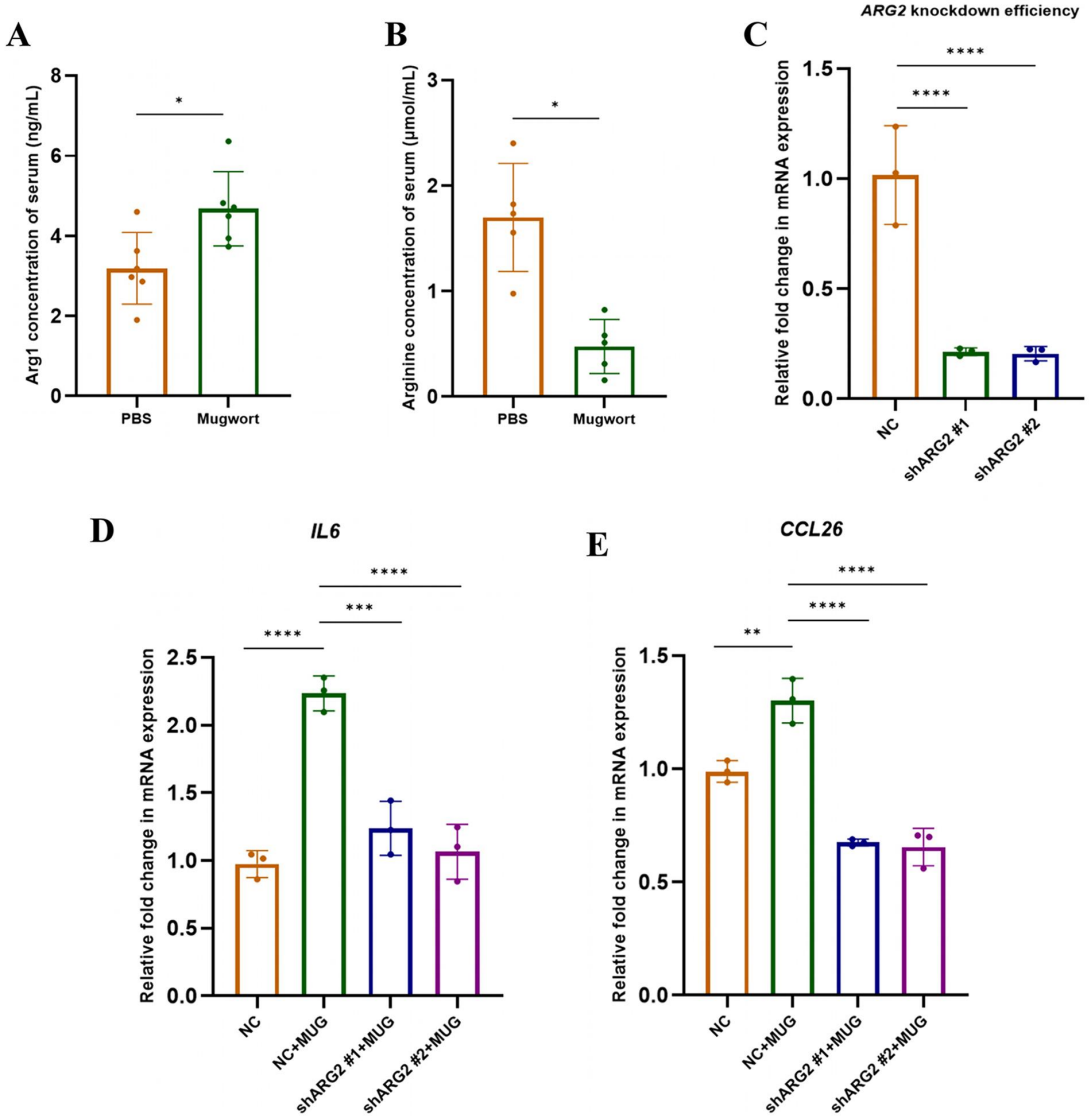
Targeted validation of arginine-related metabolic changes and ARG2-associated epithelial responses after mugwort exposure. **(A)** Levels of Arg1 in the PBS and mugwort groups measured by ELISA, *n* = 6/group. **(B)** Serum L-arginine levels in PBS and mugwort groups were measured using a visible spectrophotometric colorimetric assay, *n* = 6/group. **(C)** Knockdown efficiency of *ARG2* in BEAS-2B cells transduced with two independent shRNAs targeting *ARG2*. Relative *ARG2* mRNA expression was determined by qPCR. (D,E) Relative mRNA expression of *IL6* **(D)** and *CCL26* **(E)** in BEAS-2B cells following 200 μg/mL mugwort extract stimulation with or without *ARG2* knockdown. Data are presented as mean ± SD. Relative mRNA expression levels were normalized to *β-actin.* Statistical significance was determined using an unpaired Student's *t*-test for comparisons between two groups and one-way ANOVA for comparisons among multiple groups. *p < 0.05, **p < 0.01, ***p < 0.001, ****p < 0.0001.

To further explore the arginine-related pathways highlighted by the integrated transcriptomic-metabolomic analysis, we performed targeted validation using mouse serum samples and human epithelial cell lines. Compared with PBS controls, mugwort-treated mice showed modest but significant elevation of Arg1 levels (Figure 6A), accompanied by decreased serum L-arginine concentrations (Figure 6B). These results provide additional biochemical support for arginine-related metabolic perturbation in the allergic inflammation induced by mugwort.

We further examined whether disrupting arginine metabolism could influence epithelial responses to mugwort exposure. *ARG2*, an arginase isoform involved in intracellular arginine utilization, was selected for knockdown analysis in BEAS-2B cells. Two independent shRNAs targeting *ARG2* effectively reduced *ARG2* mRNA expression (Figure 6C). Treatment with mugwort extract at 200 μg/mL increased *IL6* and *CCL26* expression in control BEAS-2B cells, whereas this response was attenuated after *ARG2* knockdown (Figures 6D,E). These findings suggest that *ARG2* depletion partially alters the epithelial transcriptional response to mugwort extract, particularly for selected inflammatory mediators.

## 4 Discussion

Our study depicted that mugwort-induced AR is featured by coordinated activation of local inflammation, impairment of the epithelial barrier-related transcriptional program, and metabolic reprogramming. Together, these findings offered a comprehensive view of the pathological changes of this model.

Firstly, transcriptomic analysis of the nasal mucosa revealed a distinct Th2-polarized inflammatory microenvironment, characterized by the upregulation of *Il13*, *Arg1*, *Ccl24*. Integration with TF-associated network analysis suggested that the inflammatory program may be partly associated with candidate TFs such as *Fos*, *Batf*, and *Mafb*. The predominance of a coordinated transcriptional response centered on chemokine signaling networks, rather than isolated genetic changes, pointed to a paradigm shift in our understanding of mugwort-induced AR pathogenesis.

Importantly, our data revealed another pathological aspect beyond the classical immune activation. The downregulated genes were significantly enriched in pathways related to motile cilia and epithelial cell differentiation. In parallel, structural genes, such as *Krt25*, *Krt71*, and members of the *Krtap* family, were also markedly inhibited. These results suggested that mugwort exposure may be associated with changes in epithelial homeostasis-related transcriptional programs, particularly those involving ciliary function and epithelial differentiation. Since we did not directly assess mucociliary clearance or epithelial barrier function, these findings should be considered as transcriptomic evidence and require further validation.

At the metabolic level, the non-targeted serum metabolomics detected the accumulation of 1-methylhistamine, which was consistent with enhanced activation of mast cells during allergic responses. Additionally, differential metabolites were enriched in the arachidonic acid metabolism pathway, which plays an important role in inflammatory lipid mediator production (18, 19). Since eicosanoids derived from arachidonic acid metabolism are closely related to vascular permeability, mucosal edema, and nasal symptoms (20, 21), these results provided systematic biochemical support for the inflammatory phenotype observed in the AR model.

Through cross-omics integration, we further verified arginine-related metabolism as a shared altered pathway across both omics layers. Although the enriched KEGG pathway was annotated as “arginine and proline metabolism”, the mapped genes and metabolites identified in our datasets were chiefly linked to the branch of arginine metabolism. Over the past few years, arginine metabolism disturbance has gained considerable attention in the field of allergic disorders. As a non-essential amino acid in the body, arginine participates in protein synthesis and the urea cycle, serving as a precursor of multiple metabolites such as ornithine, citrulline, and nitric oxide. Studies have shown that arginine metabolism could be dysregulated during allergic inflammation in the lungs (22, 23). In our study, the presence of *Arg1* and *Arg2* among the transcriptomic hits raised the possibility that dysregulated arginine utilization was linked to the inflammatory microenvironment in the nasal mucosa, whereas alterations in metabolites such as L-arginine and spermidine pointed to broader metabolic remodeling at the systemic level.

We further measured a small-scale knockdown experiment in BEAS-2B cells. *ARG2* was selected because it is an arginase isoform involved in intracellular arginine utilization (24), and our preliminary experiments showed that *ARG2* could be more reliably knocked down in BEAS-2B cells than other candidate genes. After *ARG2* knockdown, mugwort extract-induced upregulation of *IL6* and *CCL26* was attenuated, suggesting that *ARG2* may contribute to selected epithelial inflammatory responses following mugwort exposure. It should be noted that the cell experiment was not designed to reproduce all molecular changes observed in the mouse nasal mucosa. The RNA-seq data were generated from mouse nasal mucosal tissue, whereas the qPCR validation was performed in a human epithelial cell line. *CCL26* was assessed as a human epithelial eotaxin-family chemokine in BEAS-2B cells (25, 26), but not as a direct validation target of mouse *CCL24*. This difference in model system should be considered when interpreting these *in vitro* results.

Several limitations would be considered. Firstly, this study was conducted in a mouse model, and the results may not fully reflect the complexity of human seasonal AR. Secondly, the proposed integrated regulator*y* axis is derived primarily from correlations between the transcriptome and metabolome, and therefore remains correlational in nature. Thirdly, the added serum measurements and *ARG2* knockdown experiment provide only preliminary support for the involvement of arginine-related metabolism. These data are not sufficient to define a causal role for this pathway in mugwort-induced allergic inflammation. Further studies involving arginase inhibition, metabolite supplementation, epithelial-specific genetic manipulation, or rescue experiments are still needed to determine whether arginine metabolic remodeling directly drives mugwort-induced allergic inflammation. Finally, due to limited nasal mucosal tissue availability, not all transcriptomic candidates, protein-level changes, or ciliary markers were experimentally confirmed in the present study. Despite these limitations, our findings provide additional insight into the local and systemic molecular changes associated with mugwort-induced AR and may help guide future mechanistic studies.

## Data Availability

The RNA-seq data generated in this study have been deposited in the NCBI Sequence Read Archive under BioProject accession number PRJNA1474528 and are accessible at https://www.ncbi.nlm.nih.gov/bioproject/PRJNA1474528. The remaining data supporting the conclusions of this article are included in the article and Supplementary Material. The raw metabolomics data will be made available by the corresponding author(s) upon reasonable request.
