# Supplementary material for: Artemisia pollen-induced allergic rhinitis in mice: multi-omics dissection of local and systemic molecular alterations
Source: Front Allergy. 2026 Jul 8;7:1835987. doi: 10.3389/falgy.2026.1835987 (PMC13388909; doi:10.3389/falgy.2026.1835987)
Supplement: Supplementary file 2 [file Table1.docx]

**Supplementary Table S1. Annotation confidence and differential statistics of key metabolites**

| **Annotation item** | **1-Methylhistamine** | **L-Arginine** | **Homocarnosine** | **L-Glutamic acid 5-phosphate** |
| --- | --- | --- | --- | --- |
| m/z | 126.10225 | 175.11846 | 241.12861 | 210.01646 |
| Retention time (min) | 0.57 | 0.646 | 0.585 | 0.785 |
| Ion mode | pos | pos | pos | pos |
| Annotation level | Level 1 | Level 1 | Level 2 | Level 2 |
| HMDB | HMDB0000898 | HMDB0000517 | HMDB0000745 | HMDB0001228 |
| METLIN | 5854 | 13 | 270 | 63480 |
| PubChem | 3614 | 6322 | 10243361 | 193475 |
| KEGG | C05127 | C00062 | C00884 | C03287 |
| Score | 66.8 | 69.93 | 47.24 | 48.42 |
| Isotope similarity | 72.01 | 91.25 | 75.48 | 72.43 |
| Fragmentation score | 96.86 | 91.97 | 0 | 0 |
| Fragment ions | 67.0418, 68.0495, 80.0493, 82.0649, 97.0759, 102.9482, 109.0758, 121.9940, 126.1023, 127.0498 | 60.0557, 70.0650, 72.0807, 112.0866, 116.0702, 130.0970, 157.1079, 158.0919, 159.0758, 175.1183 | 43 | 77.9688, 80.0471, 117.9872, 118.9950, 146.0184, 147.0260, 194.9934, 209.9992, 210.0166, 210.0401 |
| Adducts | M+H | M+H | M+H | M+H-H2O |
| Formula | C6H11N3 | C6H14N4O2 | C10H16N4O3 | C5H10NO7P |
| Mass error (ppm) | -3.172 | -2.855 | -3.732 | -1.428 |
| VIP | 1.635 | 1.008 | 1.293 | 0.931 |
| Average (Mugwort) | 24.402 | 25.083 | 15.858 | 22.249 |
| Average (PBS) | 23.212 | 25.576 | 16.988 | 22.643 |
| log₂ fold change | 1.19 | -0.493 | -1.131 | -0.394 |
| Fold change | 2.281 | 0.711 | 0.457 | 0.761 |
| Regulation | Up | Down | Down | Down |
| *p*-value | 1.41×10^−4^ | 0.003082 | 0.0403 | 1.39×10^−4^ |
| *q*-value | 0.0146 | 0.0425 | 0.1601 | 0.0146 |

Abbreviations: VIP, variable importance in projection.
